# Short-term effect of simulated salt marsh restoration by sand-amendment on sediment bacterial communities

**DOI:** 10.1371/journal.pone.0215767

**Published:** 2019-04-29

**Authors:** François Thomas, James T. Morris, Cathleen Wigand, Stefan M. Sievert

**Affiliations:** 1 Biology Department, Woods Hole Oceanographic Institution, Woods Hole, MA, United States of America; 2 Sorbonne Université, CNRS, Integrative Biology of Marine Models (LBI2M), Station Biologique de Roscoff (SBR), Roscoff, France; 3 Belle Baruch Institute for Marine & Coastal Sciences, University of South Carolina, Columbia, SC, United States of America; 4 U.S. EPA, Office of Research and Development, National Health and Environmental Effects Research Laboratory, Atlantic Ecology Division, Narragansett, RI, United States of America; Stazione Zoologica Anton Dohrn, ITALY

## Abstract

Coastal climate adaptation strategies are needed to build salt marsh resiliency and maintain critical ecosystem services in response to impacts caused by climate change. Although resident microbial communities perform crucial biogeochemical cycles for salt marsh functioning, their response to restoration practices is still understudied. One promising restoration strategy is the placement of sand or sediment onto the marsh platform to increase marsh resiliency. A previous study examined the above- and below-ground structure, soil carbon dioxide emissions, and pore water constituents in *Spartina alterniflora*-vegetated natural marsh sediments and sand-amended sediments at varying inundation regimes. Here, we analyzed samples from the same experiment to test the effect of sand-amendments on the microbial communities after 5 months. Along with the previously observed changes in biogeochemistry, sand amendments drastically modified the bacterial communities, decreasing richness and diversity. The dominant sulfur-cycling bacterial community found in natural sediments was replaced by one dominated by iron oxidizers and aerobic heterotrophs, the abundance of which correlated with higher CO_2_-flux. In particular, the relative abundance of iron-oxidizing *Zetaproteobacteria* increased in the sand-amended sediments, possibly contributing to acidification by the formation of iron oxyhydroxides. Our data suggest that the bacterial community structure can equilibrate if the inundation regime is maintained within the optimal range for *S*. *alterniflora*. While long-term effects of changes in bacterial community on the growth of *S*. *alterniflora* are not clear, our results suggest that analyzing the microbial community composition could be a useful tool to monitor climate adaptation and restoration efforts.

## Introduction

Salt marshes are extraordinarily productive ecosystems found in estuaries worldwide. At the interface of ocean and land, they experience shifting salinities and dynamic redox environments coupled to tidal and seasonal cycles. Salt marshes provide a variety of ecosystem services including storm protection and nutrient control. In particular, salt marsh sediments house diverse microbial communities [1,2] and are known as sites of intense cycling of nitrogen [3–8] and sulfur [9–13]. In salt marshes, the degradation of organic matter occurs predominantly through sulfate reduction, producing hydrogen sulfide which in turn fuels sulfur-oxidizing microorganisms [10,11]. The cord grass *Spartina alterniflora* is well adapted to sulfidic conditions by having its own defense mechanisms [14,15] as well as by promoting the growth of sulfur-oxidizing microorganisms in the rhizosphere [10], making it the dominant plant in areas of the USA Atlantic Coast that are submerged for parts of each tidal cycle [16].

This fine balance is being threatened by sea-level rise, which is expected to increase erosion, fragmentation, and drowning of salt marsh habitats, hence altering their productivity and biogeochemistry [17,18]. As sea level rises, the increased frequency, longer duration, and greater depths of tidal inundation will lower rates of marsh grass production [19]. This, in turn, may reduce the transfer of new photosynthates from aboveground tissues to belowground roots and rhizomes as well as the exudation of labile plant carbon into the rhizosphere. At the same time, soil redox conditions will likely become more reducing, pH levels will become lower, and pore water salinity and sulfide concentrations will increase, impeding the growth of *Spartina* [20–22] and modifying microbial metabolism. Consequently, adaptation to accelerated sea level rise with actions such as wetland restoration via sediment amendments may become necessary and more common to sustain coastal marsh resiliency [23,24]. Yet, the effects of such restoration strategies on microbial communities and marsh soil biogeochemistry are presently poorly understood.

In a recent study, Wigand et al. [24] investigated the effect of different inundation regimes on sand-amended and natural salt marsh sediments over the growing season of *S*. *alterniflora* covering a period of six months. They observed a strong influence of sand application, namely lower pH, phosphate, sulfide, ammonium, and salinity and higher CO_2_-fluxes compared to natural sediments. This contrasted with overall similar belowground productivity and biomass, suggesting that despite the changes in biogeochemical parameters restoration by sand amendment could be a viable strategy. Microbes residing in the sediment constitute a significant, yet at present inadequately understood component of the response of salt marshes to changes in sediment type and inundation regimes [25]. Here, we have analyzed the sediments at the end of the growing season at the lowest and highest elevation to examine if and how the observed changes are reflected in the bacterial communities. We hypothesized that along with the previously reported geochemical responses, sand-amendments would also influence the structure of bacterial communities.

## Material and methods

### Experimental setup and sampling

Sediment cores originated from the same experiment as described in Wigand et al. [24] (S1 Fig). The field study was conducted from April 26 to September 15, 2011 on a salt marsh at Laws Point (MA, USA, latitude 42.73, longitude -70.84) within the Plum Island Ecosystems Long Term Ecological Research (PIE-LTER) site. In Massachusetts, salt marsh land can be owned privately. Law's Point is owned by the Essex County Greenbelt who has given the PIE-LTER authority to use their land for research purposes. The placement of the mesocosms did not involve dredging filling or altering the marsh, therefore a Notice of Intent was not required under the Massachusetts wetland protection act. Briefly, a mesocosm “organ” [26] was built of an array of PVC pipes with five different heights set at the marsh edge representing different elevations spanning from 47 cm below to 17 cm above mean high water (mhw) [24]. For the present study, we only sampled pipes at the lowest (hereafter "bottom shelf") and highest (hereafter "top shelf) elevations. Pipes contained natural marsh sediment collected from a nearby creek, except for the top 40 cm that were filled with PVC inserts containing either a sand/marsh sediment mix (vol:vol 3:1; hereafter "sand-amended sediment") or natural marsh sediment. The sand was collected from a nearby quarry (Middleboro, MA) and was not further treated before mixing with marsh sediment. One field-collected *S*. *alterniflora* plug was planted into each insert on April 26. Above and below-ground plant biomass, chemical composition of pore water at 21 cm depth and carbon dioxide flux were determined as described previously [24] and data obtained in September for cores used in the present study are summarized in S1 Table. At the end of the experiment, cores were frozen at -20°C until further processing. Independent duplicate cores were retrieved for each combination of conditions (type of sediment x elevation).

### Nucleic acid extraction

Cores were thawed at 4°C and cut in half longitudinally with sterile tools. From each core, approximately 500 mg of sediment were collected at three depths, namely 1 cm, 10 cm and 21 cm, the latter corresponding to the depth of the lysimeter from which pore water was collected for chemical analysis. DNA was extracted using the Power Soil DNA isolation kit (MOBIO), following the manufacturer’s instructions. DNA yields and quality were checked by spectrophotometry on a Nanodrop 2000C (ThermoScientific).

### 16S rRNA gene tag sequencing

Library preparations and tag sequencing were performed by Molecular Research Lab (Shallowater, TX). The V1-V3 region of 16S rRNA genes was amplified using the universal bacterial primer pair 27Fmod (AGRGTTTGATCMTGGCTCAG plus unique barcodes) and 519Rmodbio (GTNTTACNGCGGCKGCTG) [27] with an *in silico* coverage of 82% for Bacteria (Silva TestPrime analysis with Silva v132) and the HotStarTaq Plus Master Mix Kit (Qiagen, Valencia, CA) as follows: initial denaturation for 3 min at 94°C, followed by 28 cycles of 94°C for 30 seconds, 53°C for 40 seconds and 72°C for 1 min, and a final elongation step at 72°C for 5 min. All PCR products from different samples were mixed in equal concentrations and purified using Agencourt Ampure beads (Agencourt Bioscience Corporation, MA, USA). Samples were sequenced on a Roche 454 FLX Titanium instrument using the manufacturer’s reagents. Raw sequence data were deposited in the SRA database under BioProject ID PRJNA507114, with sample accessions SAMN10484801 to SAMN10484824.

### 16S rRNA gene read analysis

The 16S rRNA gene sequences were processed in the QIIME 1.9.1 pipeline [28]. Reads were filtered for length (400 bp ≤ length ≤ 1000 bp), quality score (mean, >25), number of ambiguous bases (= 0) and number of homopolymer runs (<6). Chimeras detected using usearch61 *de novo* were removed from the dataset. Sequences were clustered using swarm [29] with default parameters. Clusters containing only one sequence in the full dataset (singletons) were removed. Taxonomy was assigned to each Operational Taxonomic Unit (OTU) using the RDP classifier [30] with the Silva v132 database [31]. Data were further analyzed with the package phyloseq v1.24.2 [32] in R v3.5.0 [33]. Alpha-diversity indices were calculated on a rarefied dataset at 1,524 reads per sample (smallest sequencing depth).

### Statistical analysis

Statistical analyses were performed in R v3.5.0 [33] with the packages vegan v2.5–3 [34] and phyloseq v1.24.2 [32]. Data were standardized according to the Hellinger method. The Morisita-Horn distance was used to calculate the dissimilarity matrix. Hierarchical cluster analyses was performed using the ward.D2 algorithm. Differences in bacterial assemblage structure were tested using PERMANOVA (*adonis* function with 999 permutations), testing the effect of sediment type on the global dataset and the effect of elevation and depth separately for natural and sand-amended sediments. Ordination was performed using Non Metric Multidimensional Scaling. The function *envfit* was used to fit environmental vectors onto the ordination. The following environmental parameters measured in [24] were tested on the ordination of all samples: aboveground biomass, belowground biomass, average plant height, nitrogen content, carbon content, carbon to nitrogen ratio and CO_2_ flux. Only vectors for variables with significant correlations to the ordination were plotted. Additionally, *envfit* was performed for salinity, pH, hydrogen sulfide, ammonium, and phosphate for samples collected at 21 cm, corresponding the to depth of the lysimeters. Taxa showing differential abundance between natural and sand-amended sediments were detected using DESeq2 [35] as implemented in phyloseq, with Benjamini-Hochberg correction of the p-values for multiple testing. Spearman correlation analysis, ANOVA, Mann-Whitney and Student t-tests were performed in R. OTU membership analysis was performed on the MetaCoMET web platform [36].

## Results

### Effect of sand-amendment on bacterial diversity

**Fig 1:**
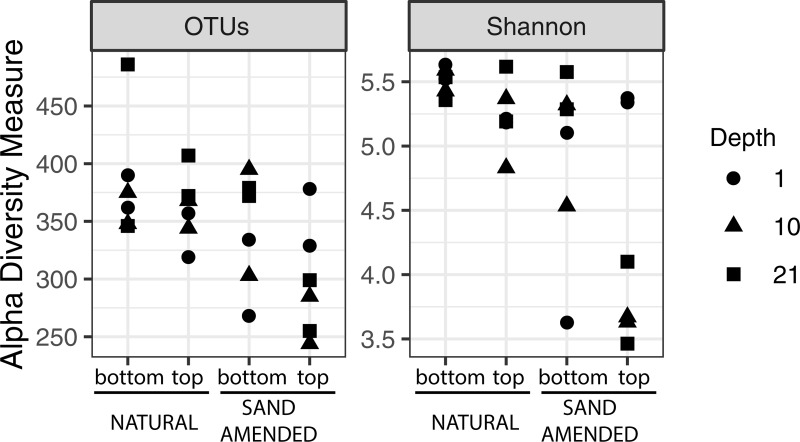
Richness (number of OTUs) and diversity (Shannon H') indices for bottom (47 cm below mean high water, mhw) and top (+17 cm above mhw) shelves in the marsh organ at 1, 10, and 21 cm depth from the sediment surface in the core mesocosms.

DNA was extracted from a total of 24 sediment samples (S2 Table), obtained from three depths at two elevations (top and bottom shelf) and two sediment types (natural or sand-amended). The yield of DNA was significantly impacted by the type of sediment (natural soil: 10.3±1.8 μg.g^-1^; sand-amended: 4.2±1.0 μg.g^-1^, t-test P<0.001) and the sample depth (1 cm: 9.8±2.6 μg.g^-1^; 10 cm: 8.3±1.7 μg.g^-1^; 21 cm: 3.7±1.2 μg.g^-1^, ANOVA P<0.001), but not by the shelf elevation (top: 7.8±2.1 μg.g^-1^; bottom: 6.8±1.2 μg.g^-1^, t-test P = 0.99). This suggests that the microbial biomass was higher in natural sediment and at the sediment surface. A total of 68,123 reads for the V1-V3 region of the 16S rRNA gene were retrieved after quality filtering and removal of singletons, ranging from 1,524 to 7,756 reads per sample. These sequences were clustered into 8,017 OTUs, with 244 to 486 OTUs per sample (S2 Table). There was a significant effect of the type of sediment (Fig 1) on both OTU richness (ANOVA, F = 8.16, P = 0.01) and Shannon diversity (F = 10.82, P = 0.004), while no significant effect was detected for elevation and depth. Natural sediment samples were both richer and more diverse than sand-amended samples.

### Community structure and effect of environmental parameters

**Fig 2:**
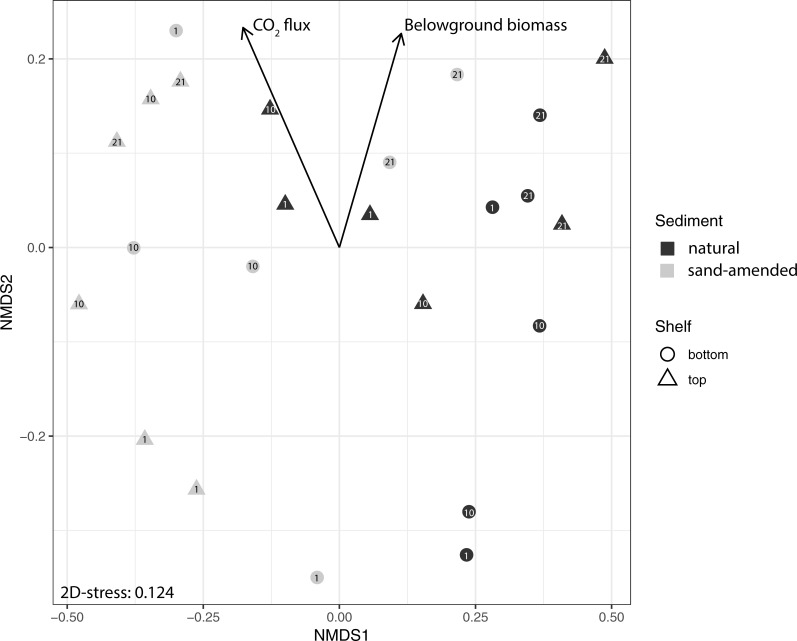
Non-metric multidimensional scaling of the bacterial community composition at the OTU level based on Bray-Curtis dissimilarity on Hellinger-transformed data. Colors depict the sediment type (black, natural sediment; grey, sand-amended sediment), shapes depict the elevation (circles, bottom shelf; triangles, top shelf) and depth is indicated by a number within the symbol for each sample (1, 10 or 21 cm from sediment surface). Environmental vectors with significant fits (p<0.05) are depicted.

**Fig 3:**
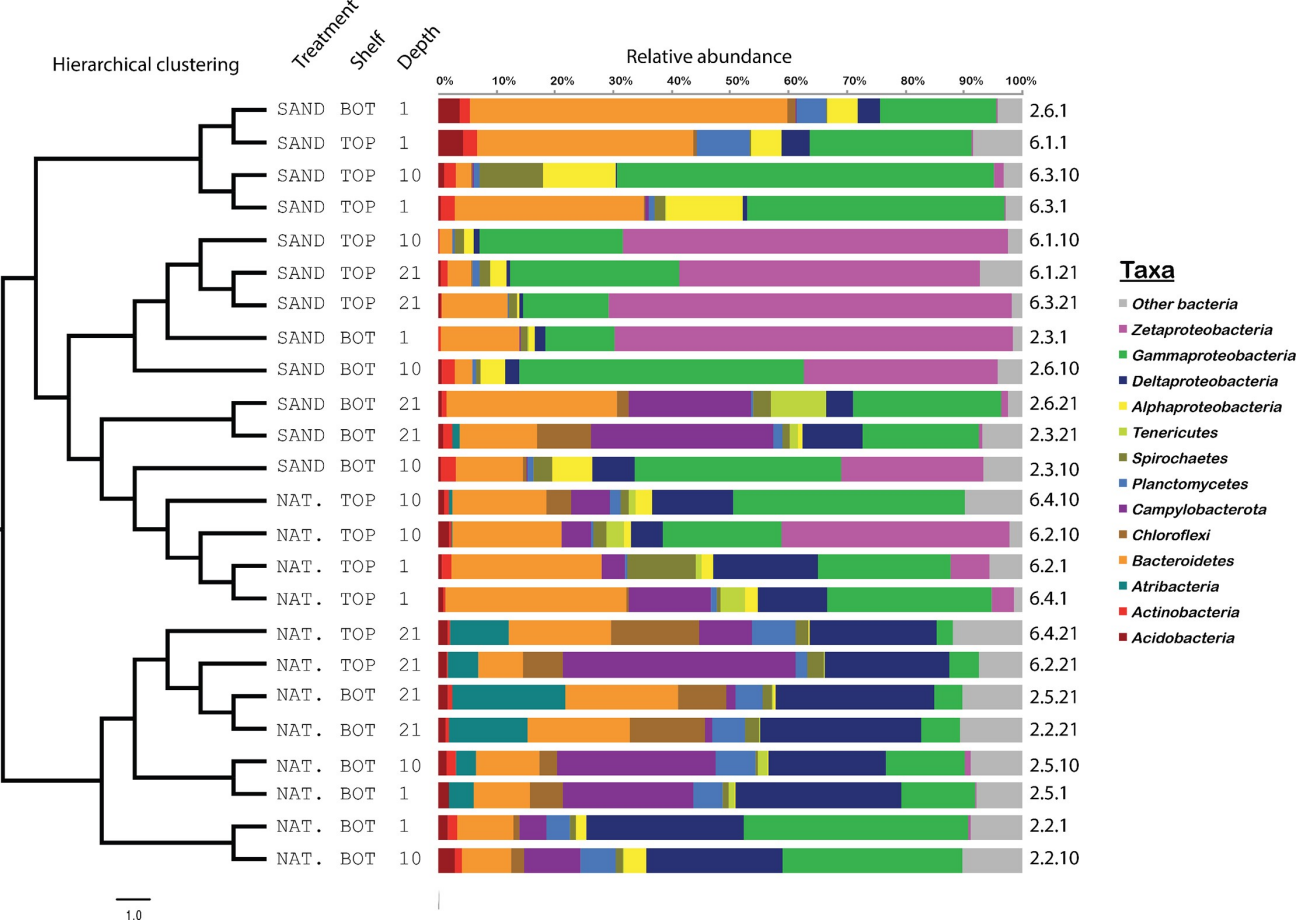
Hierarchical clustering analysis and taxonomic composition of bacterial communities from natural (NAT.) or sand-amended (SAND) sediments, positioned on the top or bottom (BOT) shelf and sampled at 1, 10 or 21 cm depth below the sediment surface. Cluster analysis was performed on a Morisita-Horn dissimilarity matrix with the Ward D2 algorithm.

The structure of bacterial communities was investigated at the OTU level. Considering all samples, there was a strong effect of the sediment type, as revealed by the NMDS ordination (Fig 2), the cluster analysis (Fig 3) and PERMANOVA (F = 1.39, p = 0.001; S3 Table). We further investigated differences according the depth and elevation, separately for natural and sand-amended sediments. PERMANOVA revealed significant differences in bacterial assemblage structure according to both elevation and depth for natural sediments, but not for sand-amended sediments (S3 Table). Overall, all natural sediments of the bottom shelf as well as the deepest layer (21 cm) of the top shelf formed a cluster (Fig 3), while the natural sediments from the upper two depths (1 cm and 10 cm) of the top shelf grouped with the sand-amended sediments, in particular with the two samples collected at 21 cm depth and one at 10 cm depth from sand-amended cores on the bottom shelf. Fitting of environmental parameters onto the NMDS ordination revealed that CO_2_-flux (r^2^ = 0.34, p = 0.02) and belowground biomass (r^2^ = 0.26, p = 0.05) were significantly correlated to the bacterial community structure. Pore water samples were only collected for the depth of the lysimeters at 21 cm [24], restricting the analysis of the effect of salinity, pH, hydrogen sulfide, ammonium, and phosphate on the bacterial communities to this depth. Only salinity (r^2^ = 0.92, p = 0.006) and pH (r^2^ = 0.71, p = 0.08) were found to be significantly correlated to the bacterial community composition at this depth.

### Effect of sand-amendment on taxonomic composition

Taxonomic affiliation of OTUs showed a total of 51 bacterial phyla in the dataset (S2 Fig). Overall, the most abundant phyla were *Proteobacteria* (55% of total reads), *Bacteroidetes* (17%) and *Campylobacterota* (8%, previously *Epsilonproteobacteria* [37,38]). The composition of the bacterial community varied between samples. Duplicate cores from the same condition showed similar taxonomic profiles at a broad taxonomic resolution (Fig 3B), with notable exceptions for three pairs (6.2.10 vs. 6.4.10; 2.3.1 vs. 2.6.1; 6.1.10 vs. 6.3.10) where the main difference was a marked variation in the relative abundance of *Zetaproteobacteria*.

**Fig 4:**
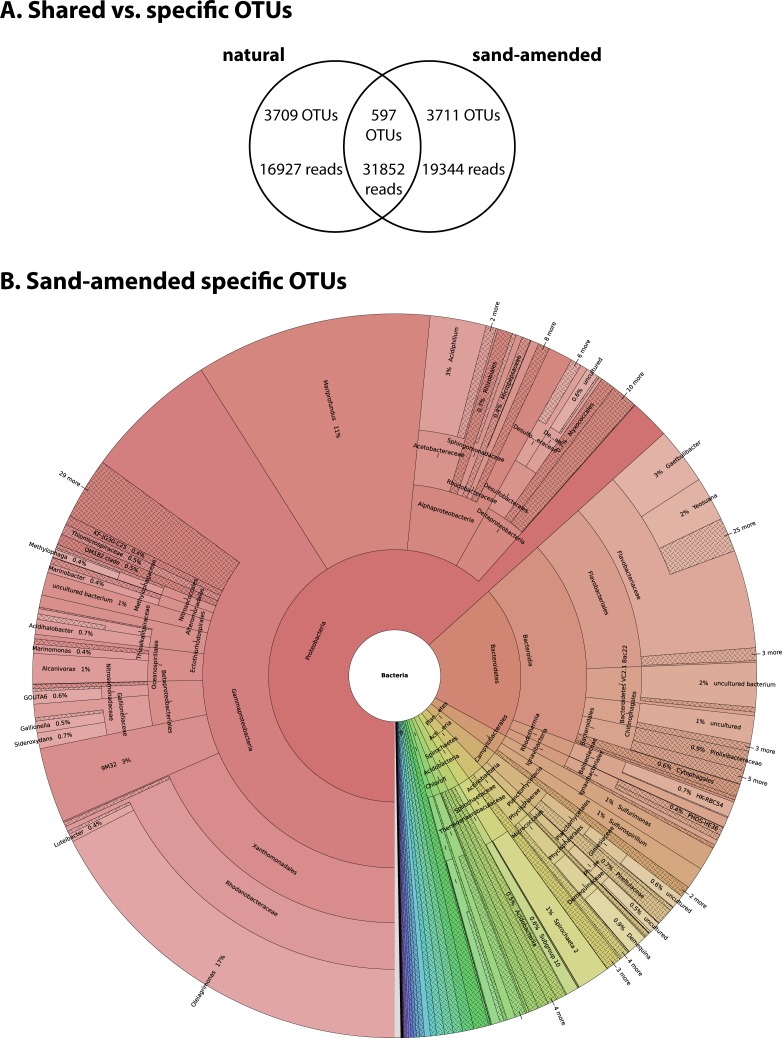
OTU membership analysis. **A.** Venn diagram showing the number of OTUs and the associated number of reads either shared by natural and sand-amended samples or specific to each condition. **B.** Taxonomic relative abundance of OTUs found exclusively in sand-amended sediments and not in natural sediments.

We first investigated the effect of sand-amendment restoration on the presence/absence of bacterial taxa at the OTU level (Fig 4A). Among the 8,017 total OTUs detected, only 7% were shared between natural and sand-amended sediments, representing 31,852 reads (47% of the total reads). On the other hand, 3,709 OTUs were only found in natural sediments, representing 16,927 reads (25% of total reads); 3,711 OTUs were specific to sand-amended sediments, representing 19,344 reads (28% of total reads). These OTUs detected only after sand addition belonged mostly to *Proteobacteria* and *Bacteroidetes* (Fig 4B). In particular, sand-amended specific OTUs showed a high diversity and relative abundance of *Mariprofundus* (514 OTUs, 2,040 reads), *Oleiagrimonas* (388 OTUs, 3,372 reads) and *Flavobacteriaceae* (289 OTUs, 2,017 reads). Half of the reads from OTUs shared between natural and sand-amended sediments belonged to only 29 *Mariprofundus* OTUs (16,912 reads), whereas other less abundant groups such as *Gammaproteobacteria* (178 OTUs, 5,260 reads), *Bacteroidetes* (153 OTUs, 3,738 reads), *Campylobacterota* (55 OTUs, 1,227 reads) and *Deltaproteobacteria* (54 OTUs, 1,518 reads) were more diverse.

**Fig 5:**
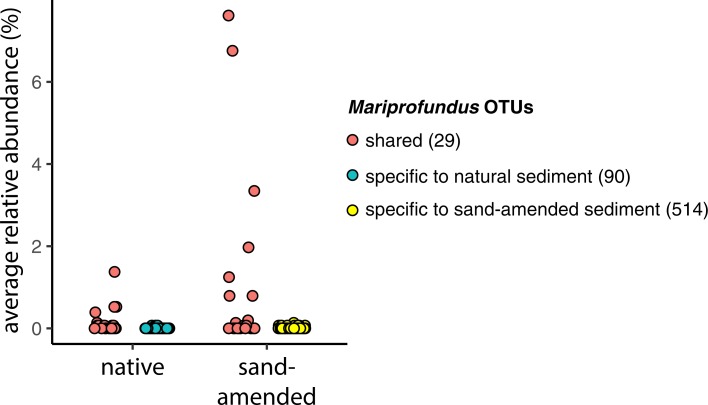
Average relative abundance of individual *Mariprofundus* OTUs that are shared (pink), specific to natural sediments (blue) or specific to sand-amended sediments (yellow). Numbers in brackets show the number of OTUs in each category.

A statistical analysis was further performed at the class and order levels to identify bacterial taxa for which the relative abundance changed according to the type of sediment (Table 1). In total, 29 taxa from 14 different classes showed a significant difference in relative abundance between natural and sand-amended sediments. Nine bacterial classes were favored in sand-amended samples compared to natural marsh sediment, the dominant ones being *Alphaproteobacteria*, *Gammaproteobacteria*, and *Zetaproteobacteria* (comprising only the genus *Mariprofundus*). The 6-fold higher abundance of *Mariprofundus* in sand-amended sediments was due to only a few individual OTUs that were shared between conditions, although there were many more sand-amended specific *Mariprofundus* OTUs (Fig 5). The effect on *Gammaproteobacteria* was mostly due to *Xanthomonadales* (Table 1), the relative abundance of which increased 13-fold between natural sediments (mean relative abundance 0.9%, range 0 to 8%) and sand-amended samples (mean relative abundance 11.5%, range 0 to 50%). Within the *Planctomycetes* phylum, sand-amendments were associated with a higher abundance of *Planctomycetacia*, but a lower abundance of *Phycisphaerae*. Furthermore, results showed a strong negative impact of sand amendment on the abundance of *Deltaproteobacteria* (6-fold decrease compared to natural sediments) and *Atribacteria* of the JS1 group (43-fold decrease) (Table 1).

**Table 1:** Global analysis of bacterial taxa showing a significant difference in relative abundance between natural and sand-amended sediments. The analysis was performed at the Class and Order levels. Only taxa with a Benjamin-Hochberg corrected p-value lower than 0.05 are shown. For each taxon, bold values represent the condition with the highest relative abundance.

Phylum	Class	Order	Mean relative abundance (%)	
			Natural sediment	Sand- amended
*Acidobacteria*	*Acidobacteriia*	*-*	0.01	**0.22**
*Actinobacteria*	*Actinobacteria*	*-*	0.25	**0.96**
*Actinobacteria*	*Actinobacteria*	*Micrococcales*	0.10	**0.63**
*Actinobacteria*	*Thermoleophilia*	*-*	0.03	**0.21**
*Atribacteria*	*JS1*		**4.72**	0.11
*Bacteroidetes*	*Bacteroidia*	*Chitinophagales*	0.07	**1.01**
*Cloacimonetes*	*Cloacimonadia*	*-*	**0.21**	0.01
*Cyanobacteria*	*Oxyphotobacteria*	*-*	**0.22**	0.12
*Patescibacteria*	*Saccharimonadia*	*-*	0.01	**0.44**
*Patescibacteria*	*Saccharimonadia*	*Saccharimonadales*	0.01	**0.44**
*Planctomycetes*	*Phycisphaerae*	*-*	**3.09**	0.51
*Planctomycetes*	*Phycisphaerae*	*mle1-8*	**0.21**	0.00
*Planctomycetes*	*Phycisphaerae*	*MSBL9*	**2.29**	0.17
*Planctomycetes*	*Planctomycetacia*	*-*	0.36	**1.27**
*Planctomycetes*	*Planctomycetacia*	*Planctomycetales*	0.01	**0.73**
*Proteobacteria*	*Alphaproteobacteria*	*-*	1.26	**4.51**
*Proteobacteria*	*Alphaproteobacteria*	*Acetobacterales*	0.04	**2.30**
*Proteobacteria*	*Deltaproteobacteria*	*-*	**20.47**	3.18
*Proteobacteria*	*Deltaproteobacteria*	*Desulfuromonadales*	**2.16**	0.17
*Proteobacteria*	*Deltaproteobacteria*	*Sva0485*	**0.25**	0.00
*Proteobacteria*	*Gammaproteobacteria*	*-*	18.76	**30.49**
*Proteobacteria*	*Gammaproteobacteria*	*Acidithiobacillales*	0.00	**1.87**
*Proteobacteria*	*Gammaproteobacteria*	*EPR3968-O8a-Bc78*	0.10	**0.22**
*Proteobacteria*	*Gammaproteobacteria*	*KI89-clade*	**0.03**	0.00
*Proteobacteria*	*Gammaproteobacteria*	*OM182-clade*	0.04	**0.36**
*Proteobacteria*	*Gammaproteobacteria*	*Xanthomonadales*	0.87	**11.49**
*Proteobacteria*	*Zetaproteobacteria*	*-*	4.28	**26.33**
*Verrucomicrobia*	*Verrucomicrobiae*	*-*	0.07	**0.32**
*Verrucomicrobia*	*Verrucomicrobiae*	*Opitulales*	0.01	**0.27**

### Potential effect on community functions

**Fig 6:**
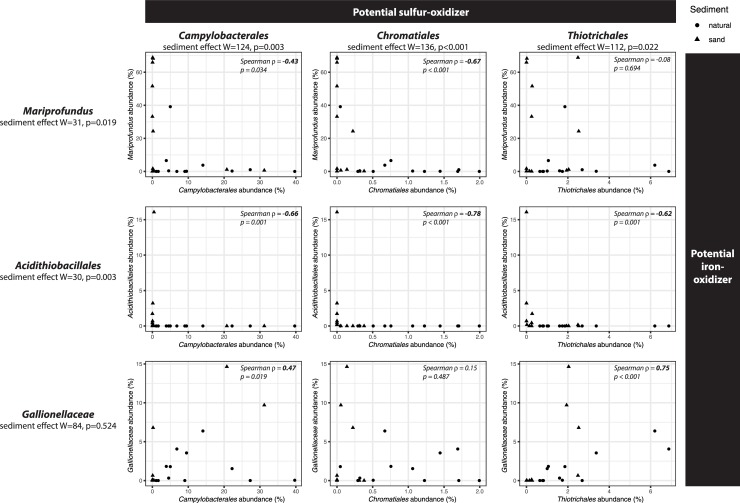
Effect of sand-amendments and correlation analysis on the relative abundance of potential sulfur-oxidizer taxa and potential iron-oxidizing taxa in natural (circles) and sand-amended (triangles) sediments. For each taxon individually, the effect of sediment type was tested using Wilcoxon test. For each pair of taxa, the Spearman correlation ρ and the associated p-value are reported. Values in bold denote statistical significance (p<0.05).

A previous study of cores from the same experiment reported significantly lower pore water pH and sulfide concentrations in sand-amended sediments [24], suggesting a shift between bacterial communities with contrasting metabolic capabilities. Therefore, we further analyzed the abundance of selected bacterial groups potentially involved in iron or sulfur oxidation, processes known to influence pH and sulfide concentrations in salt marshes (Fig 6). For sulfur cycling, we considered potential S-oxidizers in the orders *Chromatiales*, *Thiotrichales* and *Campylobacterales*, recently shown to be prevalent in *S*. *alterniflora*-vegetated salt marsh sediments [10]. In natural sediments, the relative abundance of *Campylobacterales* ranged from 1 to 40%, exceeding that for *Chromatiales* (0.05–2%) and *Thiotrichales* (0.6–7%). This is in contrast with previous results obtained for vegetated sediments on a bank ca. 500 m away from our present study site, where *Campylobacterales* accounted less than 1% of 16S rDNA Illumina reads [10]. These three potential S-oxidizer orders showed a significantly lower abundance in sand-amended sediments compared to natural sediments (Fig 6 "sediment effect", Mann-Whitney test, p<0.05). The most drastic decrease was observed for *Campylobacterales*, which in 10 out of 12 sand-amended samples did not reach more than 0.44% relative abundance. The only exceptions were sand-amended samples from 21 cm depth on the bottom shelf (31% and 20% *Campylobacterales* for samples 2.3.21 and 2.6.21, respectively) that were already shown to resemble natural sediments on the NMDS ordination plot and cluster analysis (Figs 2 and 3). For iron cycling, we considered as potential Fe-oxidizers the taxa *Mariprofundus*, *Acidithiobacillales* and *Gallionellaceae* (formerly known as the order-level taxon *Gallionellales*, but reclassified as a family in SILVA v132 [39]). Both *Mariprofundus* and *Acidithiobacillales* were favored in sand-amended sediments compared to natural sediments, whereas no significant sediment effect was detected for *Gallionellaceae* (Fig 6). There were sharp negative correlation patterns for the abundance of *Mariprofundus* with *Campylobacterales* and *Chromatiales*, and for *Acidithiobacillales* with the three potential S-oxidizer groups. Communities with high relative abundance of S-oxidizer groups tended to have low relative abundance of Fe-oxidizer groups, and *vice versa*. The position of the points, close to the x and y axis of the scatter plot, suggests that these bacterial groups involved in sulfur and iron cycling were almost mutually exclusive. By contrast, positive correlations were found for the abundance of *Gallionellaceae* with *Campylobacterales* and *Thiotrichales* (Fig 6, bottom row). In particular, the two samples with the highest *Gallionellaceae* abundance (10 and 15%) were the same sand-amended samples from 21 cm depth on the bottom shelf with high abundance of *Campylobacterales*.

Another key finding of the previous study on the cores from the same experiment was the increase of soil carbon dioxide emission in the sand-amended mesocosms [24], putatively linked to more aerobic conditions and higher organic matter decomposition than in natural sediments. Here, we evaluated the associations of the relative abundance of bacterial orders with CO_2_ flux and carbon content, used as a proxy for the amount of organic matter in the sediments. CO_2_ emission was positively correlated with the abundance of *Mariprofundales* and unclassified *Gammaproteobacteria* (Table 2). In addition, *Mariprofundales* were correlated negatively to the sediment carbon content. Furthermore, the potential sulfate-reducing deltaproteobacterial orders *Desulfarculales* and *Desulfobacterales* were associated positively with carbon content, together with *Anaerolinales* (phylum *Chloroflexi*) and the candidate *Phycisphaerae* order MSBL9 (phylum *Planctomycetes*).

**Table 2:** Correlations of the abundance of order-level taxa with CO_2_ flux and carbon content. The Spearman ρ statistics is given, together with the associated p-value in brackets. Bold values denote significance at p<0.1. Orders accounting more than 1% of the total number of reads were tested, and only those with at least one significant correlation are reported.

Order	Spearman correlation with	
	CO_2_ flux^a^	Carbon content^b^
*Anaerolineales*	-0.31 (p = 0.46)	**0.39 (p = 0.06)**
*Phycisphaerae* MSBL9	-0.44 (p = 0.27)	**0.43 (p = 0.03)**
*Acetobacterales*	0.32 (p = 0.44)	**-0.41 (p = 0.04)**
*Desulfarculales*	-0.40 (p = 0.33)	**0.40 (p = 0.05)**
*Desulfobacterales*	-0.36 (p = 0.39)	**0.49 (p = 0.01)**
Unclass. *Gammaproteobacteria*	**0.67 (p = 0.08)**	0.03 (p = 0.90)
*Mariprofundales*	**0.76 (p = 0.04)**	**-0.39 (p = 0.06)**

^a^ Calculated with the average values across 3 depths per core.

^b^ Calculated with local values at each depth.

## Discussion

Salt marshes provide essential ecosystem services, yet their existence is threatened by accelerated sea level rise, making it imperative to find ways to build marsh resiliency. One possible mitigation strategy that has gained attraction in the Northeast USA is placement of sediment or sand (including beneficial re-use of dredged material) onto the marsh platform to build elevation, which can reduce flooding duration and optimize marsh plant productivity [40,41]. Organic matter accumulation in the marsh soils, associated with plant productivity, will help the marsh keep pace with sea level rise. However, the effects of this restoration treatment on resident microbial communities are presently not well understood. Here, we report on bacterial community responses to different inundation periods and sand enrichment to coastal marsh soils. This study is part of a larger study that examined the responses of marsh above-and below-ground structure and processes to sand amendments at varying inundations regimes at five elevations [24]. Although we only sampled at the end of the experiment and only the lowest and highest elevations, we believe our results reflect the bacterial community changes that could be expected under similar initial conditions due to manipulation by sand-amendment and by a prolonged inundation regime, as we have assessed the two extreme flooding cases.

As previously shown, our results clearly indicate a prevalence of sulfur-metabolizing bacteria in the natural salt marsh sediments [2,10,12], which also constituted the dominant taxa of the shared microbiome between the natural and sand-amended sediments. Sulfate-reducing *Deltaproteobacteria* and sulfur-oxidizing *Gammaproteobacteria* and *Campylobacteria*, along with *Bacteroidetes*, dominated the microbial communities in the natural sediments, in particular the lower elevations and deeper sediment layers known to be submerged the longest, which consequently also had measurable sulfide [24]. The only exception here was a sample from 10 cm depth at the highest elevation (Fig 3B, sample 6.2.10), which showed a much higher relative abundance of potential iron-oxidizing *Zetaproteobacteria*. This could possibly be related to the absence of any measurable sulfide throughout the growth season in this particular core [24]. This contrasts with all other samples, including a sample from the same depth of a replicate core (6.4.10), and it might have tilted the conditions from sulfide oxidation in favor of iron oxidation. The very high sulfide levels at 21 cm in the lowest elevation (S1 Table) are probably a result of the high abundance of sulfate-reducing bacteria combined with the lower abundance of sulfur-oxidizing bacteria, creating conditions that might not be favorable for *S*. *alterniflora*. Here, we found a generally higher abundance of *Campylobacteria* compared to a previous study on a nearby site at PIE-LTER [10]. Since the primers used to create the sequencing libraries have similar predicted coverage for *Campylobacteria* (94% against V1-V3 in this study; 97% against V6 region in [10]), PCR bias is unlikely to be the main cause for the observed difference. This could rather be due to the position of the present experimental cores closer to the creek bank, resulting in more frequent flooding and overall more reducing conditions (i.e., higher sulfide and lower oxygen), that might favor *Campylobacteria* over *Gammaproteobacteria* [42,43], reflecting the spatial heterogeneity in salt marshes. In addition, the confinement within the core liners might have created conditions different from the natural surrounding sediment. The positive correlation of carbon content with the abundance of the deltaproteobacterial orders *Desulfarculales* and *Desulfobacterales*, *Anaerolinales* and the candidate *Phycisphaerae* order MSBL9 (phylum *Planctomycetes*) [44,45] is most likely related to the less rapid degradation of organic matter in the more anoxic, organic-rich natural sediments and sequestration of belowground plant material (i.e., roots and rhizomes).

Along with the observed changes in biogeochemistry described by Wigand et al. [24], sand amendments led to drastic changes in bacterial community composition, with only 7% of the observed OTUs being shared with natural sediment. However, these shared OTUs belonged to the more abundant taxa, as they represented 47% of the total reads. Interestingly, *Mariprofundus* accounted for a large portion of the shared community, even though they generally represented only a relatively small component of the natural sediment community, indicating their ability to respond quickly to changing conditions. On the other hand, natural sediment taxa were lost upon sand amendments, as communities became less rich and less diverse. This could possibly result in a loss of function, with potentially negative effects to the plant. In general, the microbial community in sand-amended sediments switched from a sulfur-cycling dominated community described above to one dominated by potential iron oxidizers and aerobic carbon degradation. This way, sand amendment had a strong negative impact on anaerobic bacteria such as *Atribacteria* of the JS1 group [46] and *Deltaproteobacteria*. This is not unexpected due to the more oxidizing conditions in the sand amended conditions [24]. In parallel, microorganisms with a putative aerobic metabolism increased, such as *Alphaproteobacteria*, *Gammaproteobacteria*, and *Zetaproteobacteria*. While *Alphaproteobacteria* and *Gammaproteobacteria* are expected to be aerobic heterotrophs and thus contribute to the increased CO_2_-flux, the *Zetaproteobacteria* are Fe-oxidizing chemolithoautotrophs which might benefit from the increased CO_2_ and oxygen levels and the decreased sulfide levels, making them more competitive compared to sulfur-oxidizing bacteria. This becomes very obvious when comparing the relative abundance of sulfur-oxidizing bacteria and the Fe-oxidizer zetaproteobacterial *Mariprofundus*, with the former clearly favored in the natural sediments and the latter in the sand-amended sediments (Fig 6). Interestingly, sand amendments appear to boost the growth of only a few *Mariprofundus* OTUs that are already present in natural marsh sediment, even though the added sand introduced many additional novel OTUs (Fig 5). Therefore, this drastic change in microbial community structure is driven more by changes in environmental conditions than by the introduction of allochthonous microorganisms. The low abundance of potential sulfur-oxidizing bacteria in sand-amended sediments is most likely a consequence of (i) the decreased sulfide production due to the lower abundance of sulfate-reducing bacteria and (ii) in case of *Campylobacteria* the possibly higher oxygen concentration. It is interesting that the potential Fe-oxidizer *Gallionellaceae* did not follow the same pattern as *Mariprofundus* (Fig 6). This suggests that *Gallionellaceae* might inhabit a different niche within these sediments, being able to oxidize iron under conditions unfavorable to *Mariprofundus* or maybe even oxidizing reduced sulfur compounds [47]. Wigand et al. [24] speculate that the oxidation of sulfide in sand-amended treatments at high elevation contributed to the lower pH. However since we did not observe a high abundance of bacterial sulfide-oxidizers, we propose that besides abiotic chemical reactions, such as pyrite (FeS_2_) oxidation [41,48], acidification may have been facilitated by Fe-oxidizing *Zetaproteobacteria* favored after sand addition. Some bacterial iron oxidizers can facilitate pyrite oxidation even at circumneutral pH [49], and recently a novel strain *Mariprofundus* sp. strain GSB2 was isolated from an iron-oxide mat at pH 6.2 in a New-England salt marsh that can grow on FeS [50]. This suggests that Fe-oxidizing *Zetaproteobacteria* observed in the organ cores containing natural sediments might also play a role in the oxidation of iron sulfides in natural salt marsh sediments. *Mariprofundus* isolates feature a filamentous stalk-like structure composed of iron oxyhydroxides, the formation of which produces acidity according to the following equation: Fe_3_^+^ + 3H_2_O -> Fe(OH)_3_ + 3H^+^ [51]. Combined with a lower buffering capacity, this could explain the lower pH observed in sand-amended sediments compared to natural sediments. Similarly, the accumulation of iron oxides was associated with a sharp decrease of the pH in a lowland rice rhizosphere [52]. Further, the significantly lower pore water phosphate in the sand-amended soil [24] may also be a result of bacterially mediated iron oxidation, e.g., by *Zetaproteobacteria*, and adsorption of phosphate to iron-oxyhydroxides. There was also a significant higher ammonium concentration in the natural sediments of the bottom shelf compared to sand-amended sediments. This has previously been attributed to a reduced ammonium uptake by *S*. *alterniflora* under the more sulfidic conditions in the natural sediments [24,53,54]. However, in addition the anaerobic conditions could have led to an increased production of ammonium, for example due to dissimilatory reduction of nitrate to ammonium (DNRA) [8,55], and/or lower consumption due to aerobic ammonium oxidation. At the same time, aerobic ammonium oxidation could have been enhanced in the sand-amended sediments. While we did not observe ammonium-oxidizing bacteria in these sediments, ammonium-oxidizing archaea that were not targeted in our metabarcoding analysis could have been present [7].

While the sand-amendment clearly had the strongest effect on the bacterial communities, we also observed an effect of elevation and sediment depth in natural sediments, but not in sand-amended sediments. This might be attributed to the increased porosity of the sand-amended sediments, likely resulting in more homogenous and generally more aerobic conditions due to increased flushing [24]. However, the communities in the deepest layer of the sand-amended sediments on the bottom shelf showed similarities to natural sediments on the top shelf at the end of the growing season (Figs 2 and 3). This suggests that the community structure might eventually equilibrate if the optimal growth conditions for *S*. *alterniflora* are maintained. To facilitate the establishment of a community that is more similar to natural conditions, a possible strategy could be to mix the sand with a higher proportion of natural sediment. *S*. *alterniflora* is known to grow in niches with high sulfide concentrations (0.5–1 M) that are inhibitory to other wetland plants [56,57]. Thus, generally lower sulfide concentrations in sand-amended sediments could lead to increased competition with other emergent plants, including natural, invasive, and non-natural ones that might colonize the disturbed marsh soils and possibly replace *S*. *alterniflora*. Along these lines, the significantly higher abundance of *Xanthomonadales* bacteria could be a sign for general unhealthy conditions in the sand-amended sediments of the upper shelf. Indeed, previous studies showed that *Xanthomonadales* present in low abundance in natural salt marsh sediments increased significantly after fertilization with nitrate [58], indicating their opportunistic growth response. Furthermore, although that has not been described specifically for *S*. *alterniflora*, many *Xanthomondales* are known as phytopathogens [59] that might affect plant growth. At present, it is not clear what would be the long-term effects (> 5 months) of a significantly changed bacterial community as seen in the sand-amended sediments at higher elevations on the growth of *S*. *alterniflora*. While detrimental effects could not be excluded, the results of the deeper layers of the sand-amended sediments at lower elevations indicate that communities might eventually equilibrate, provided the inundation regime is within the optimal range for *S*. *alterniflora*.

## Supporting information

S1 Fig Natural and sand-amended mesocosms with inserts and lysimeters, and elevation levels labelled. (TIF)

S2 Fig Phylum-level taxonomic distribution of all sequences retrieved during this study (singleton-free, n = 68,123). "Others" comprises phyla accounting less than 1%.
(PNG)

S1 Table Summary of above and belowground plant biomass, carbon dioxide flux, and chemical composition of pore water at 21 cm depth. (PDF)

S2 Table Description of the samples and alpha-diversity measures. (PDF)

S3 Table Summary of permutational analysis of variance (PERMANOVA with 999 permutations) testing the difference in bacterial community structure according to sediment source (on all samples together) and elevation and depth (separately for natural and sand-amended sediments). (PDF)
