# Supplementary figures and images for: Short-term effect of simulated salt marsh restoration by sand-amendment on sediment bacterial communities
Source: PLoS One. 2019 Apr 29;14(4):e0215767. doi: 10.1371/journal.pone.0215767 (PMC6488055; doi:10.1371/journal.pone.0215767)

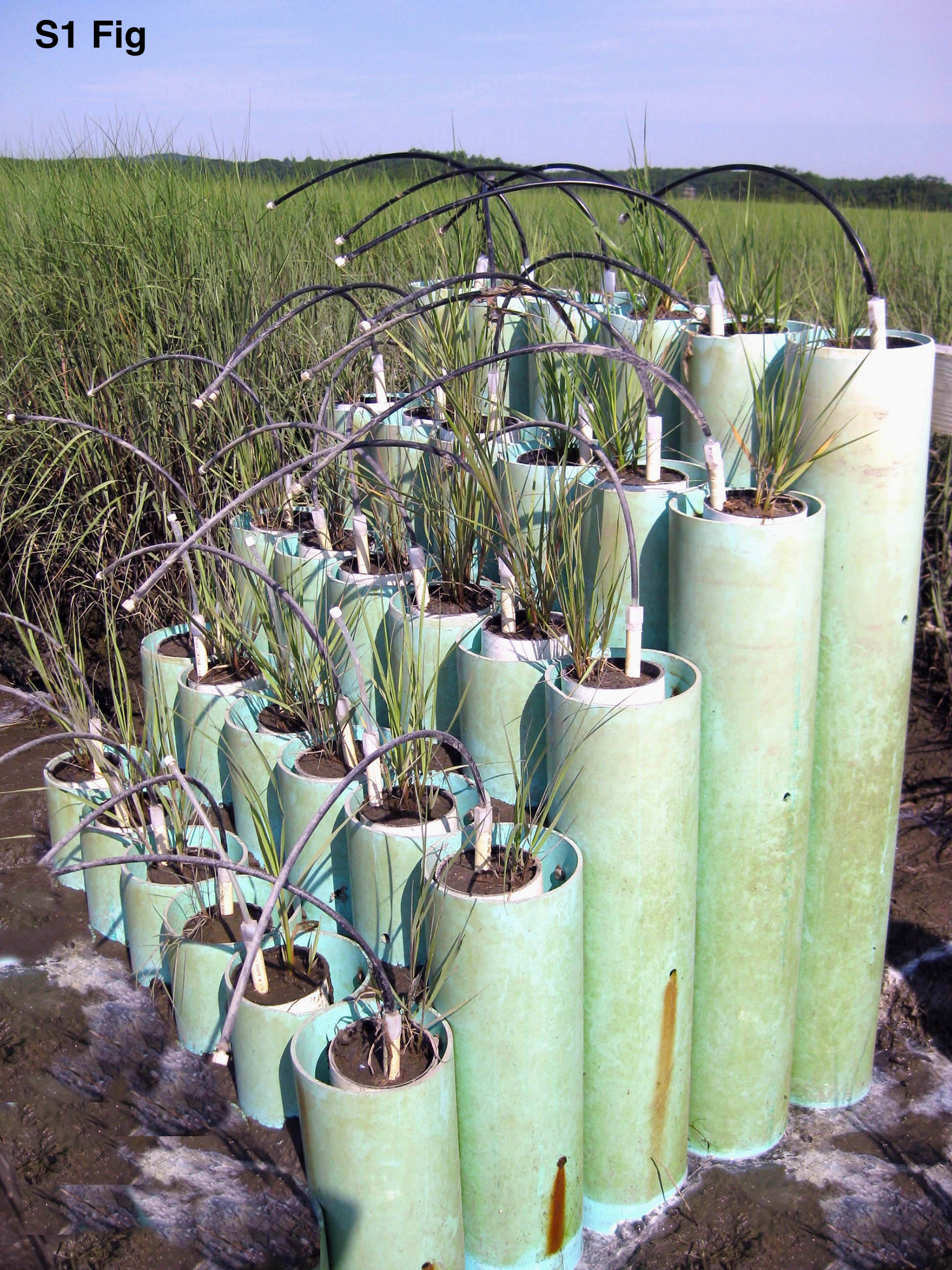

Supplement: S1 Fig — (TIF) [file pone.0215767.s001.tif]

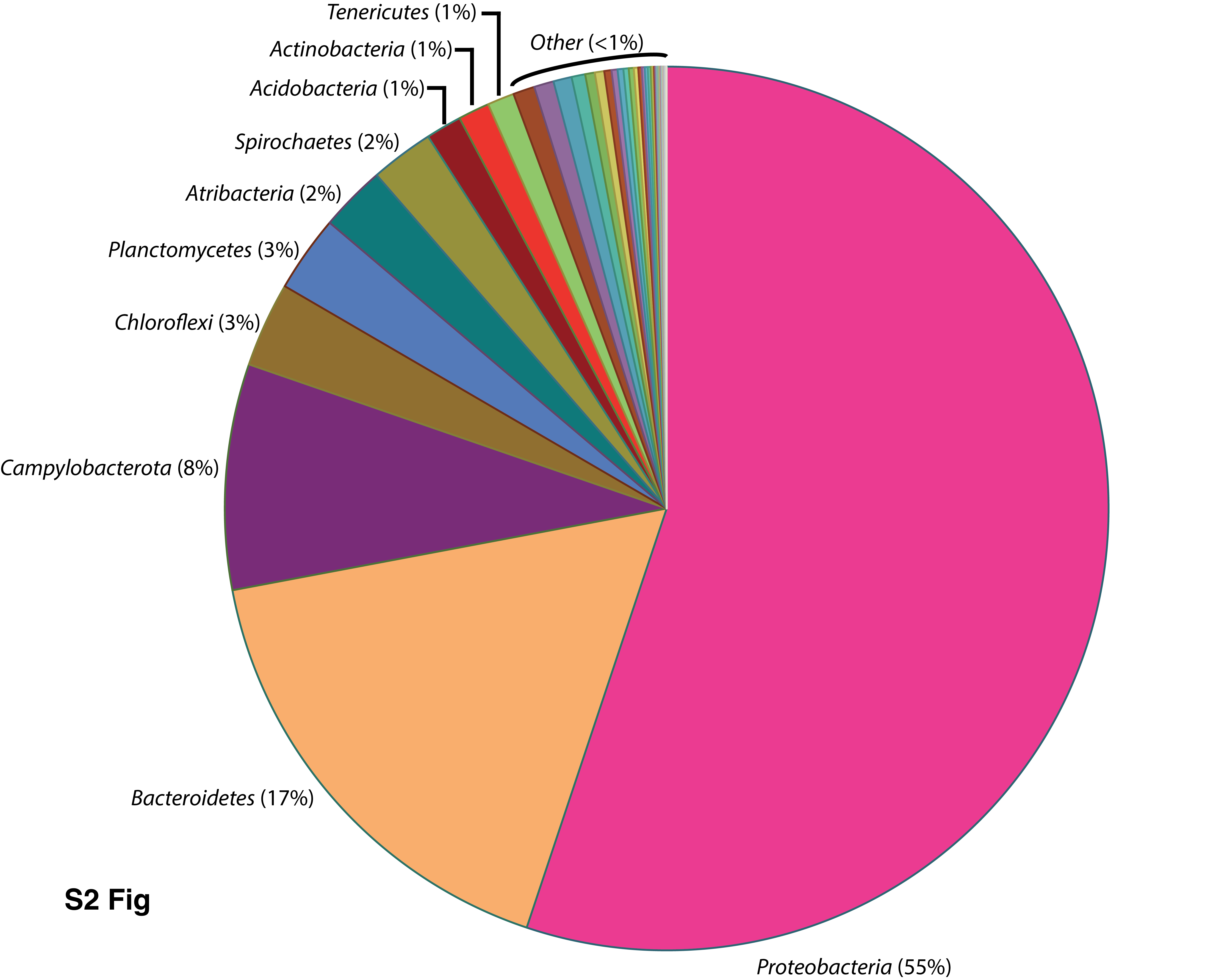

Supplement: S2 Fig — "Others" comprises phyla accounting less than 1%. (PNG) [file pone.0215767.s002.png]
