# Supplementary material for: Short-term effect of simulated salt marsh restoration by sand-amendment on sediment bacterial communities
Source: PLoS One. 2019 Apr 29;14(4):e0215767. doi: 10.1371/journal.pone.0215767 (PMC6488055; doi:10.1371/journal.pone.0215767)
Supplement: S1 Table — (PDF) [file pone.0215767.s003.pdf]

S1 Table: Summary of above and belowground plant biomass, chemical composition of pore water at 21 cm depth and carbon dioxide flux.

	Natural sediment		Sand-amended	
	**Bottom**	**Top**	**Bottom**	**Top**
**CO_2 flux (μmol CO_2.m^{-2}.s^{-1})**	1.36	3.47	3.07	3.82
**Aboveground (g.m^{-2})**				
Dead	31.1	127.3	50.0	47.3
Alive	324.9	400.0	269.5	318.7
Total	356.0	527.3	319.5	366.0
**Belowground biomass (gdw.m^{-2})**	3762.1	6255.8	3170.7	3354.7
**Carbon content (C %)**	3.95	3.56	1.53	2.95
**Nitrogen content (N %)**	0.30	0.27	0.12	0.23
**C/N ratio**	12.95	13.35	12.03	14.76
**Salinity (ppt)**	30.0	29.5	24.0	20.5
**pH**	7.40	6.40	5.74	5.80
**H_2S (μM)**	80.1	0.0	20.8	0
**PO_4 (μM)**	77.5	0.14	2.39	0.43
**NH_4 (μM)**	337.5	2.4	5.5	2.2

Values are mean of two independent replicate cores. Adapted from Wigand *et al*. [24]
