# Supplementary material for: Short-term effect of simulated salt marsh restoration by sand-amendment on sediment bacterial communities
Source: PLoS One. 2019 Apr 29;14(4):e0215767. doi: 10.1371/journal.pone.0215767 (PMC6488055; doi:10.1371/journal.pone.0215767)
Supplement: S2 Table — (PDF) [file pone.0215767.s004.pdf]

S2 Table: Description of the samples and alpha-diversity measures.

Sample ID	Sediment	Shelf	Depth (cm)	DNA yield (µg/g)	Reads	OTUs	Shannon
2.2 1	natural	bottom	1	25.2	1875	390	5.63
2.2 10	natural	bottom	10	17.5	1661	375	5.59
2.2 21	natural	bottom	21	9.0	1704	346	5.36
2.5 1	natural	bottom	1	4.8	1697	362	5.52
2.5 10	natural	bottom	10	10.5	1524	348	5.43
2.5 21	natural	bottom	21	5.0	2692	486	5.54
2.3 1	sand	bottom	1	2.4	5129	268	3.63
2.3 10	sand	bottom	10	4.0	2694	395	5.32
2.3 21	sand	bottom	21	1.3	1856	379	5.58
2.6 1	sand	bottom	1	6.6	2106	334	5.10
2.6 10	sand	bottom	10	6.1	2801	303	4.53
2.6 21	sand	bottom	21	0.6	2158	372	5.29
6.2 1	natural	top	1	12.4	1984	319	5.21
6.2 10	natural	top	10	4.7	4249	368	4.83
6.2 21	natural	top	21	5.5	2362	372	5.19
6.4 1	natural	top	1	9.6	1789	357	5.18
6.4 10	natural	top	10	12.9	1773	344	5.37
6.4 21	natural	top	21	6.8	2047	407	5.62
6.1 1	sand	top	1	12.3	1601	329	5.37
6.1 10	sand	top	10	6.0	6751	285	3.63
6.1 21	sand	top	21	0.9	4739	299	4.10
6.3 1	sand	top	1	4.7	2497	378	5.34
6.3 10	sand	top	10	4.6	2678	244	3.67
6.3 21	sand	top	21	0.8	7756	255	3.46

The three digits of sample IDs are as follows: 1st digit: elevation (2=bottom, 6=top); second digit: unique identifier for the pot number for each elevation; third digit: sampling depth (1, 10 or 21 cm). The 1st and 2nd digit are the same as the ID provided in the supplemental table of Wigand *et al*. (24).
