# Supplementary material for: Short-term effect of simulated salt marsh restoration by sand-amendment on sediment bacterial communities
Source: PLoS One. 2019 Apr 29;14(4):e0215767. doi: 10.1371/journal.pone.0215767 (PMC6488055; doi:10.1371/journal.pone.0215767)
Supplement: S3 Table — (PDF) [file pone.0215767.s005.pdf]

S3 Table: Summary of permutational analysis of variance (PERMANOVA with 999 permutations) testing the effect of sediment source, elevation and depth on the bacterial community composition.

Sample set	Source	Df	SS	MS	Pseudo-F	R^2	P(perm)
**All**	Sediment	1	0.656	0.656	1.387	0.059	0.001
	Residuals	22	10.403	0.473		0.941	
	Total	23	11.059			1.000	
**Natural**	Shelf	1	0.548	0.548	1.219	0.106	0.021
	Depth	2	1.039	0.520	1.156	0.200	0.020
	Residuals	8	3.597	0.450		0.694	
	Total	11	5.184			1.000	
**Sand-amended**	Shelf	1	0.505	0.505	1.092	0.097	0.137
	Depth	2	0.992	0.496	1.073	0.191	0.134
	Residuals	8	3.700	0.462		0.712	
	Total	11	5.197			1.000	
